# The Direct Assignment Option as a Modular Design Component: An Example for the Setting of Two Predefined Subgroups

**DOI:** 10.1155/2015/210817

**Published:** 2015-01-15

**Authors:** Ming-Wen An, Xin Lu, Daniel J. Sargent, Sumithra J. Mandrekar

**Affiliations:** ^1^Department of Mathematics, Vassar College, 124 Raymond Avenue, Poughkeepsie, NY 12604, USA; ^2^Emory University, Atlanta, GA 30322, USA; ^3^Mayo Clinic, Rochester, MN 55905, USA

## Abstract

*Background*. A phase II design with an option for direct assignment (stop randomization and assign all patients to experimental treatment based on interim analysis, IA) for a predefined subgroup was previously proposed. Here, we illustrate the modularity of the direct assignment option by applying it to the setting of two predefined subgroups and testing for separate subgroup main effects. *Methods*. We power the 2-subgroup direct assignment option design with 1 IA (DAD-1) to test for separate subgroup main effects, with assessment of power to detect an interaction in a post-hoc test. Simulations assessed the statistical properties of this design compared to the 2-subgroup balanced randomized design with 1 IA, BRD-1. Different response rates for treatment/control in subgroup 1 (0.4/0.2) and in subgroup 2 (0.1/0.2, 0.4/0.2) were considered. *Results*. The 2-subgroup DAD-1 preserves power and type I error rate compared to the 2-subgroup BRD-1, while exhibiting reasonable power in a post-hoc test for interaction. *Conclusion*. The direct assignment option is a flexible design component that can be incorporated into broader design frameworks, while maintaining desirable statistical properties, clinical appeal, and logistical simplicity.

## 1. Introduction

The primary goal of phase II clinical trials is to better understand a treatment's efficacy and safety profile to inform a phase III go/no-go decision. The phase II design with option for direct assignment (i.e., stop randomization and assign all patients to the experimental arm based on one or two interim analyses (IA)) for a single predefined subgroup was previously proposed [1]. In theory, such a design can be readily incorporated into existing and broader design frameworks. Specifically, the option for direct assignment can be integrated into any design with an IA where a decision must be made for how to allocate treatment to patients (typically the decision is between continuing to randomize patients to one of the treatments and stopping the trial due to either efficacy or futility). In this paper, we present the direct assignment option as a modular design component by applying it to the setting of two predefined subgroups.

In some therapeutic settings, we may expect treatment heterogeneity across subpopulations identified by some factor, for example, biomarker status. Specifically, the treatment may be effective in one subgroup but ineffective in another (qualitative treatment-subgroup interaction), or the treatment may be effective in both subgroups but with different magnitude (quantitative treatment-subgroup interaction). In either case, primary interest may be in both subgroups, and the design should enroll patients in both subgroups into a single trial. Such a design could allow for prospective planning of a design to identify predictive markers. For example, KRAS mutations were identified in a retrospective analysis to be predictive for overall survival response to cetuximab in colon cancer [2]. We could imagine having instead proposed a prospective phase II direct assignment design enrolling two subgroups to enable the KRAS discovery in phase II. Alternatively in some settings, primary interest may only be in one of the subgroups. However, there may be secondary interest in the second subgroup, and depending on available resources and the clinical setting, one may wish to enroll the second subgroup as well. One design option for this setting is a stratified balanced randomized design. This design could be readily modified to incorporate and enjoy the benefits of the direct assignment option introduced in An et al. [1].

In this paper therefore, we consider a design with direct assignment option for the setting of two predefined subgroups of patients. The proposed design is a 2-subgroup direct assignment design with 1 IA (DAD-1), enrolling the subgroups in parallel, each using a DAD-1 with the options to stop early for futility, continue with randomization, continue with direct assignment, or stop early for efficacy at IA (Table 1). We power the design to test for separate subgroup treatment effects, where the direction of treatment effect is prespecified, and also include a post-hoc assessment of power to detect a treatment-subgroup interaction. We compare the 2-subgroup DAD-1 with a 2-subgroup balanced randomized design with 1 IA (BRD-1), with options to stop early for futility, continue with randomization, or stop early for efficacy at IA. We perform a simulation study to examine the statistical properties of the designs, under a variety of response rate settings. Finally, we discuss a planning exercise for enrolling a second subgroup, when primary interest is only in one subgroup.

## 2. Methods

### 2.1. When Both Subgroups Are of Primary Interest

We consider a binary outcome, such as response, as the trial endpoint, and two patient subgroups, which we call M+ and M−. Since we are interested in separate treatment effects in the two subgroups, we consider two independent primary hypotheses. Let Δ_+_ and Δ_−_ denote the ratio of response rates for treated versus control groups (i.e., treatment effect) in the M+ and M− subgroups, respectively. We assume that Δ_+_ is positive (i.e., treatment is beneficial) and assume that Δ_−_ is specified a priori as either positive or negative by the investigator based on expert knowledge. Thus in the M+ subgroup, we are interested in the one-sided test H_0+_: Δ_+_ = 1 versus H_1+_: Δ_+_ > 1; in the M− subgroup, we are also interested in a one-sided test H_0−_: Δ_−_ = 1 versus H_1−_: Δ_−_ > 1 or H_0−_: Δ_−_ = 1 versus H_1−_: Δ_−_ < 1, depending on whether Δ_−_ is specified a priori to be greater or less than 1. We make no assumption on the relationship of treatment effect between the two subgroups. Specifically, we do not assume that the treatment will be beneficial in the M− subgroup only if it is first beneficial in the M+ subgroup. Since this is the phase II setting, a treatment-subgroup interaction is typically not of primary interest. However, we include an assessment of power to detect a treatment-subgroup interaction in a post-hoc test: H_0,Int_: Δ_+_/Δ_−_ = 1 versus H_0,Int_: Δ_+_/Δ_−_ ≠ 1.

We specify a desired treatment effect size Δ_*i*_, (*i* = + or −) for each subgroup, an acceptable type I error rate (*α*; the probability of rejecting the subgroup-specific null, when the null is true), and the desired power to detect the treatment effect size Δ_*i*_ (i.e., 1 − *β*, the probability of rejecting the subgroup-specific null, when the alternative Δ_*i*_ is true). We assume common *α* and *β*, but possibly different Δ_*i*_, for the two subgroups. Sample sizes are calculated separately in each subgroup. Specifically, we calculate sample size based on a one-sided 2-sample test for proportions for a 2-stage design with 1 : 1 randomization, 1 interim analysis, and O'Brien-Fleming stopping rules using EAST software, as in the original design with direct assignment option (for details, see [1]). 

*Simulation Study*. To understand the statistical properties of the 2-subgroup direct assignment option design with 1-IA (DAD-1), we conducted a simulation study. For testing the subgroup main effects, we specified 1 − *β* = 0.80 and *α* = 0.20, corresponding to widely recommended and accepted standards in the phase II setting [3]. In the M+ subgroup, we considered a control arm response rate of 0.2 versus a treatment arm response rate of  0.4. That is, we assume that the treatment is effective in the M+ subgroup (Δ_+_ = 2.0), based on preliminary studies. In the M− subgroup, we consider a control arm response rate of 0.2 versus treatment arm response rates of 0.1, 0.2, and 0.4, reflecting 3 possible scenarios: a reverse treatment effect (Δ_−_ = 0.5), no treatment effect (Δ_−_ = 1), and a treatment benefit (Δ_−_ = 2.0), respectively. Note that, here, a treatment arm response rate in the M− subgroup of either 0.1 or 0.2 corresponds to a treatment-subgroup interaction. The resulting sample size calculations are summarized in Table 2.

We simulated 500 trials for each of the two cases (Table 2). In Case I (no interaction), we consider a treatment effect in both subgroups (i.e., treatment versus control response rates: 0.4 versus 0.2, in both M+ and M−); in Case II (interaction), we consider a treatment effect in M+ (treatment versus control response rates: 0.4 versus 0.2) and a reverse treatment effect in M− (treatment versus control response rates: 0.1 versus 0.2). For each trial, we tested main effects separately in each subgroup and an interaction effect and recorded the results for testing each of the three independent hypotheses: one-sided tests for main effects—H_0+_: Δ_+_ = 1 versus H_1+_: Δ_+_ > 1; H_0−_: Δ_−_ = 1 versus H_1−_: Δ_−_ > 1 or H_1−_: Δ_−_ < 1 (depending on the a priori hypothesized treatment effect) and the two-sided test for an interaction effect—H_0,Int_: Δ_+_/Δ_−_ = 1 versus H_0,Int_: Δ_+_/Δ_−_ ≠ 1. For the post-hoc test for interaction, we used a conservative *α*-level of 0.10 (two-sided) for rejecting the null hypothesis of no interaction. Averaging these outcomes over the 500 simulated trials, we obtained estimates of type I error rate and power for each hypothesis. For comparison, we also studied the outcomes under a 2-subgroup balanced randomized (1 : 1) design with 1 IA and no option for direct assignment (BRD-1), based on the O'Brien-Fleming stopping rules.

### 2.2. When One Subgroup Is of Primary Interest: Prospective Planning Exercise for Enrolling a Second Subgroup

In some settings, interest may be only in one subgroup, say M+. However, instead of altogether excluding the other subgroup, M−, if resources are available, a design could include the M− subgroup (as long as there are no safety or efficacy concerns) by accruing to the M− subgroup while the M+ subgroup is accruing. An exploratory analysis in the M− subgroup could then yield preliminary indication of treatment effect in the M− subgroup.

To decide between enrolling M+ patients only and additionally enrolling the M− subgroup as an exploratory companion group, a prospective planning simulation exercise could be conducted. Since the M− subgroup is not of primary interest, it is likely that there is no precise preliminary information about the treatment effect in the M− subgroup. Instead, one might specify in the M− subgroup the response rate in the control arm (RR_control_) to be uniformly distributed over some interval [RR_*L*,control_, RR_*U*,control_] and the response rate ratio comparing treated versus control arms (RRR_trt:control_) to be uniformly distributed over another interval [RRR_*L*,trt:control_, RRR_*U*,trt:control_]. If the interval for the RRR_trt:control_ includes 0, then such a specification would allow for the treatment to have a negative (RRR_trt:control_ < 0), neutral (RRR_trt:control_ = 0), or positive (RRR_trt:control_ > 0) effect in the M− subgroup, thus reflecting vague knowledge about the treatment activity in the M− subgroup. It is possible then to simulate a trial and record the observed difference in response rates. Averaging across the simulated trials, one can obtain a probability of observing a difference in response rates that exceeds some threshold, say *δ*, that is of clinical interest. Of course this probability will depend on the sample size. Since accrual to M− will occur while accrual to M+ is open, the sample size for M− will depend on the accrual rates to both M+ and M− and the prevalence of the subgroups and may not be known in advance. Probabilities that the difference exceeds *δ* can be generated, under a variety of plausible settings. The investigator can use the probability distributions as a guide to decide whether to enroll the M− subgroup as an exploratory companion group, depending on whether he or she believes the probability to be sufficiently high to make enrollment into M− worthwhile.

As an example of the prospective planning exercise, we considered *δ* = 0.15. That is, an observed difference in response rates comparing treated versus control of 15% would be considered clinically relevant. We further specified the control group response rate to be uniformly distributed over [0.1,0.3] and RRR_trt:control_ to be uniformly distributed over [0.5,1.5]. We considered sample sizes of *N*_1,−_ = 6, 16, and 48 patients for the first stage in the M− group. These correspond to M+/M− prevalence of *N*_1,+_/6, *N*_1,+_/16, and *N*_1,+_/48, assuming a sample size of *N*_1,+_ in the first stage for the M+ group. For each sample size, we simulated 500 trials. We created histograms of the observed treatment differences and recorded the proportion of trials where the absolute observed treatment difference in response rates exceeded *δ* = 0.15.

## 3. Results

### 3.1. When Both Subgroups Are of Primary Interest

The nominal power and type I error rate are preserved in the 2-subgroup DAD-1, relative to a 2-subgroup BRD-1 (Table 3). In particular, for the M+ group, the power to detect a RRR_trt:control_ of 2 for DAD-1 is 78.4% (versus 78.8% for a BRD-1), and the type I error rate is 23.8% (versus 20.6% for a BRD-1). For the M− group, the power to detect a RRR_trt:control_ of 0.5 for the DAD-1 is 82.8% (versus 84.4% for a BRD-1), and the type I error rate is 19.0% (versus 18.2% for a BRD-1).

We were also interested in the properties of a post-hoc test for an interaction effect. Type I error rate is preserved at the nominal rate, and power decreases slightly relative to the nominal rate. Specifically, for Case I (no interaction), the type I error rate is 11.3% for the DAD-1, compared with 11.4% for a BRD-1. For Case II (interaction), the power to detect an interaction effect at a two-sided alpha level of 0.10 for 2 versus 0.5 is 64.3% for the DAD-1, compared with 67.6% for a BRD-1.

### 3.2. When One Subgroup Is of Primary Interest: Prospective Planning Exercise for Enrolling a Second Subgroup

**Figure 1:**
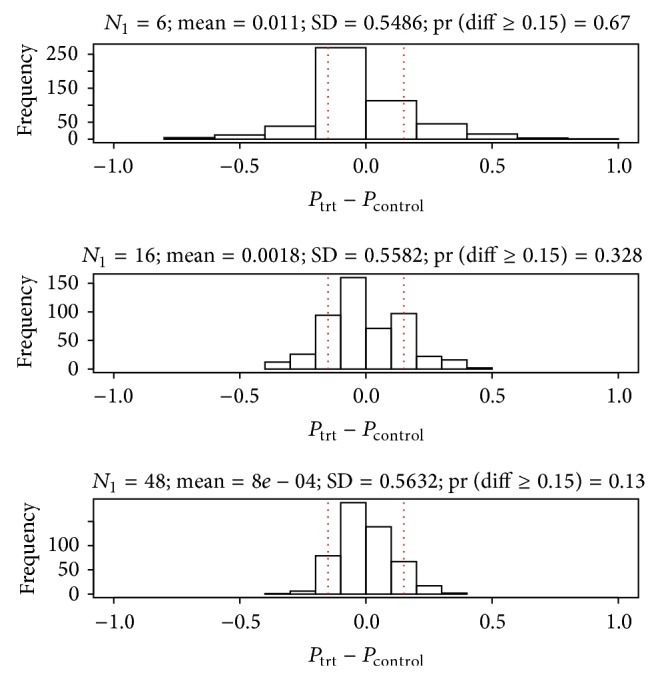
Distributions of observed treatment differences in the M− subgroup at interim analysis, across 500 simulated trials, using control response rate uniformly distributed over [0.1,0.3] and response rate ratio for treated versus control arms uniformly distributed over [0.5,1.5]. Sample sizes in the first stage, *N*_1_ = 6, 16, and 48. Proportion of trials where observed treatment difference exceeds *δ* = 0.15 in absolute value is 67%, 32.8%, and 13%, respectively.

From the 500 simulated trials, when [RR_*L*,control_, RR_*U*,control_] = [0.1,0.3] and [RRR_*L*,trt:control_, RRR_*U*,trt:control_] = [0.5,1.5], the probability of the absolute observed difference exceeding *δ* = 0.15 is 67% for *n* = 6 patients per treatment arm in the first stage (Figure 1). In contrast, using *n* = 16 (32) patients per arm in the first stage, the probability of the observed difference exceeding *δ* = 0.15 is 32.8% (13%).

## 4. Discussion

The direct assignment option design was first proposed as a design enrolling a single cohort [1]. We have applied the design in the 2-subgroup setting. The 2-subgroup DAD-1 preserves power and type I error rates at their nominal levels. Further, this design has reasonable power for planning purposes for a post-hoc test for detecting a treatment-subgroup interaction. The first finding is to be expected since the 2-subgroup DAD-1 applies the direct assignment option design in parallel to each subgroup. However, the second result that there is reasonable (post-hoc) power to detect a treatment-subgroup interaction is previously unexplored and is of potential interest.

The assessment of power to detect an interaction in a post-hoc test using the 2-subgroup DAD-1 and an *α*-level of 0.10 suggests reasonable power (64.3% to detect a response rate of 2.0 in M+ versus response rate of 0.5 in M−). We recognize that this *α*-level is conservative for an interaction test when the main effects use a one-sided *α*-level of 0.20. The sample size that was used to detect the interaction was larger than that of a typical phase II trial (65 patients in M+ and 161 patients in M−). However, the reality is that no phase II trial can reliably detect interaction effects of the size explored in this simulation study using small sample sizes. In fact, an alternative strategy of prospectively planning for an interaction test would have similarly yielded a large sample size (214 total patients, based on a balanced randomized design with no IA). Our strategy therefore has no sample size disadvantage in detecting an interaction effect relative to one that prospectively plans for a similarly sized interaction effect in a phase II setting.

The 2-subgroup DAD-1 could be applied in any setting where there are two subpopulations of interest. A natural setting is that of targeted, biomarker-based therapies. Other so-called integrated biomarker designs have previously been proposed. We highlight a couple of such designs; comprehensive reviews of such designs are available elsewhere (e.g., [4, 5]). The parallel subgroup-specific design [6] evaluates treatment effects separately in two subgroups. When the treatment effect is homogeneous across subgroups, this design has less power for detecting a treatment benefit, compared to a design that tests for an overall treatment effect. In an attempt to address the lack of power in a homogeneous treatment setting, other designs have adopted a sequential testing strategy. Specifically, the Marker Sequential Test (MaST) design first tests for a treatment effect in the M+ subgroup [7]. If this test is statistically significant, then the M− subgroup is tested. However, if the test is not statistically significant, then an overall population is tested. MaST increases power to detect treatment benefit when the treatment effect is homogeneous across subgroups and preserves power when the treatment is effective in the M+ subgroup but not the M− subgroup, as compared to a parallel subgroup-specific design.

At first glance, the proposed design resembles a parallel subgroup-specific design in spirit. As noted in Freidlin et al. [7], under the case of homogeneous treatment effect, the parallel subgroup-specific design lacks power relative to a test for overall treatment effect and the MaST design. However, the proposed design differs from the setting considered by Freidlin et al. [7] in two important ways. First, the proposed design is for the phase II setting and not the phase III setting. At this early exploratory stage, it is important to first understand the treatment effect in each subgroup separately without necessarily examining overall treatment effects. That is, although a test for overall treatment effect may be more powerful than separate tests for subgroup-specific treatment benefit when the treatment effect is homogeneous across subgroups, an overall treatment effect would not be of primary interest in the phase II setting. Second, the MaST design's property of improving power in the homogeneous treatment setting rests on a key assumption: if the treatment does not work in the M+ subgroup, then it cannot work in the M− subgroup. We refer to this as the* treatment monotonicity* assumption and do not invoke this assumption for the proposed design setting. Rather, we allow for the subgroup-specific treatment effects to be independent of each other.

It is not always the case that primary interest is in two subgroups. However, even if primary interest is only in one subgroup, it may be informative to enroll a second subgroup as an exploratory companion group (if ethical) in early phase trials. The decision to enroll a second subgroup will depend on the tradeoff among available resources, the clinical importance of the difference in response rates *δ*, and the estimated probability of the observed difference exceeding *δ*. The results from the prospective planning exercise, therefore, could aid the study design team in this decision.

## 5. Conclusion

In summary, we have illustrated at a practical level a 2-subgroup design with direct assignment option for the phase II setting where primary interest is in separate subgroup treatment effects. Such a design not only preserves the nominal type I error rate and power for testing for separate subgroup treatment effects but also enjoys reasonable post-hoc power to detect a treatment-subgroup interaction, if one exists, as well as clinical appeal and logistical simplicity. In the case where primary interest is only in one subgroup but resources may be available to enroll the second subgroup, we have proposed a planning exercise for aiding the study design team in deciding to enroll the second subgroup, using the framework of the direct assignment option design.

## Figures and Tables

**Table 1:** Options available at interim analysis (IA) in a 2-subgroup direct assignment design with 1 IA (DAD-1) versus a 2-subgroup balanced randomized design with 1 IA (BRD-1). The decisions are independently made in each subgroup at the time of IA. Some cells in the BRD-1 table are intentionally left blank, to highlight the missing option of direct assignment in this design. The options in bold are those that are available only in the design with direct assignment option.

Options at interim analysis (IA)			
2-subgroup direct assignment design with 1 IA (DAD-1)		2-subgroup balanced randomized design with 1 IA (BRD-1)	
M−	M+	M−	M+
Stop, futility	Stop, futility	Stop, futility	Stop, futility
	Continue, randomize		Continue, randomize
	**Continue, direct**		
	Stop, efficacy		Stop, efficacy

Continue, randomize	Stop, futility	Continue, randomize	Stop, futility
	Continue, randomize		Continue, randomize
	**Continue, direct**		
	Stop, efficacy		Stop, efficacy

**Continue, direct**	**Stop, futility**		
	**Continue, randomize**		
	**Continue, direct**		
	**Stop, efficacy**		

Stop, efficacy	Stop, futility	Stop, efficacy	Stop, futility
	Continue, randomize		Continue, randomize
	**Continue, direct**		
	Stop, efficacy		Stop, efficacy

**Table 2:** Sample size calculations for 1-sided *α* = 0.20 and 1 − *β* = 0.80, for different treatment effects in the two subgroups. RR_trt_ is the response rate in the treatment group, RR_control_ is the response rate in the control group, and RRR_trt:control_ is the ratio of response rates in the treatment versus control groups (i.e., treatment effect).

Case	M+ subgroup			M− subgroup		
	Treatment effect, RRR_trt:control_	RR_trt_/RR_control_	*N*	Treatment effect, RRR_trt:control_	RR_trt_/RR_control_	*N*
I (no interaction)	2	0.4/0.2	65	2	0.4/0.2	65
II (interaction)	2	0.4/0.2	65	0.5 (i.e., reverse benefit)	0.1/0.2	161

**(a):** 

Separate subgroup main effects								
Case	M+ subgroup				M− subgroup			
	Power		Type I error rate		Power		Type I error rate	
	DAD-1	BRD-1	DAD-1	BRD-1	DAD-1	BRD-1	DAD-1	BRD-1
I (no interaction)	78.4	78.8	23.8	20.6	79.8	81.8	20.8	18.6
II (interaction)	78.4	78.8	23.8	20.6	82.8	84.4	19.0	18.2

**(b):** 

Subgroup-treatment interaction effect				
Case	Power		Type I error rate	
	DAD-1	BRD-1	DAD-1	BRD-1
I (no interaction)	—	—	11.3	11.4
II (interaction)	64.3	67.6	—	—
